# Possible mechanism of *Vitis vinifera* L. flavones on neurotransmitters, synaptic transmission and related learning and memory in Alzheimer model rats

**DOI:** 10.1186/s12944-018-0708-6

**Published:** 2018-07-04

**Authors:** Lijuan Ma, Hui Xiao, Juan Wen, Zhan Liu, Yi He, Fang Yuan

**Affiliations:** 1 College of Basic Medical, Xinjiang Medical University, No.393, Xinyi Road, Urumqi, 830011 Xinjiang People’s Republic of China; 2 College of Public Health, Xinjiang Medical University, Urumqi, 830054 People’s Republic of China; 3 College of HouBo, Xinjiang Medical University, Karamay, 834000 People’s Republic of China

**Keywords:** Alzheimer disease, Flavones, *Vitis vinifera* L., Synaptic plasticity, Amyloid-β_(25–35)_, Neurotransmitter

## Abstract

**Background:**

This study explored the possible mechanism of flavones from *Vitis vinifera* L. (VTF) on neurotransmitters, synaptic transmission and related learning and memory in rats with Alzheimer disease (AD).

**Methods:**

The researchers injected amyloid-β_(25–35)_ into the hippocampus to establish AD model rats. The Sprague-Dawley (SD) rats were divided into a control group, a donepezil group, an AD model group, a VTF low-dose group, a VTF medium-dose group and a VTF high-dose group. The researchers detected the activity of choline acetyltransferase (ChAT) and acetylcholinesterase (AChE) according to kit instructions. The protein expression of brain-derived neurotrophic factor (BDNF), synaptotagmin-1 (SYT1) and cyclic adenosine monophosphate response element binding protein (CREB) in the rats’ hippocampi was detected by immunohistochemistry and Western blot, and the gene expression of cAMP-regulated enhancer (CRE) was detected by real-time quantitative polymerase chain reaction (PCR).

**Results:**

VTF may enhance the protein expression of p-CREB, BDNF and SYT1 in rat hippocampi, depending on dose. The messenger RNA (mRNA) level of CREB was significantly higher in the VTF high-dose group than in the model group, which was consistent with the results of Western blotting. VTF may reduce the activity of AChE and increase that of ChAT in rat hippocampi. Finally, VTF effectively improved the learning and memory abilities of AD rats.

**Conclusions:**

VTF can promote synaptic plasticity and indirectly affect the expression of cholinergic neurotransmitters, which may be one mechanism of VTF protection in AD rats.

## Background

CREB can regulate spatial learning and memory to maintain normal function of synaptic plasticity; thus, it is often referred to as “a molecular switch that regulates memory”. The synaptic plasticity–related proteins BDNF and SYT1 can not only promote neuronal regeneration but also regulate synaptic plasticity, thereby affecting the learning and memory functions of the brain. *BDNF* and *SYT1* are the downstream target genes of CREB. Phosphorylated CREB (p-CREB) promotes the survival and regulation of synaptic plasticity and closely regulates these downstream genes in nerve cells [1–3]. The cholinergic hypothesis suggests that the decrease in synthesis of the neurotransmitter acetylcholine (ACh) is one of the most important causes of cognitive impairment, which is the most significant symptom of AD in neurochemical pathology [4].

In both animal and clinical experiments, the researchers found that consumption of plants and fruits rich in flavonoid compounds can repair injured neurons, thereby enhancing memory [5]. The study found that flavonoids can have a protective effect on neurons similar to that of neurotrophic factors and can also delay brain lesions [6, 7]. Flavonoids from *Ginkgo biloba* extract can improve neurotoxic damage to hippocampal neurons [8], while total flavonoids from *Pueraria lobata* can activate CREB and promote production of BDNF [9].

Aβ has been shown to cause neurotoxicity [10, 11].Aβ is the root cause of AD. Aggregation of Aβ plays an important role in AD pathogenesis [12, 13].In this study, the rat model of AD was established by Aβ_25-35_, which was the core toxic fragment of Aβ_1-42_, as an ideal animal model for AD. We hypothesed that VTF improved the learning and memory functions by effecting neurotransmitters and synaptic plasticity. The aim of the study was to test the hypothesis.

## Methods

### Animals

A total of 90 male SD rats (7–8 weeks; 250 ± 20 g) were purchased from the Animal Center of Xinjiang Medical University (quality certificate no. 650007000). All animal experiments were conducted according to the ethical guidelines of Xinjiang Medical University. All efforts were made to minimize animal suffering.

### Sample preparation

#### Preparation of aggregated Aβ_(25–35)_ peptide

Aβ_(25–35)_ was dissolved in deionized water to a concentration of 1 mmol/L and incubated at 37 °C for 7 days. After aggregation, the sample was stored at 4 °C.

#### Preparation of flavones from *Vitis vinifera* L.

*V. vinifera* L. was purchased from an Uyghur medicine market in Turpan. The researchers dried and smashed *V. vinifera* L. grapes, then performed 3 separate extractions lasting 2 h each using a 95% ethanol solution. The extracted solution was merged for rotary evaporation until there was no alcohol taste. Flavonoids were extracted with water after suspension, then purified with AB-8 macroporous resin and a 5% water/95% ethanol gradient elution. The researchers then collected a 50% ethanol elution fraction from the flavonoids and vacuum dried it at 60 °C, thus obtaining *V. vinifera* L. (VTF) flavones in the form of a brown-yellow powder.

### Establishment of AD model

The AD model was established in the rats after 1 week of normal feeding in a specific-pathogen-free (SPF) laboratory. The rats were anesthetized using 3.0% sodium pentobarbital. The model group, the donepezil group (positive control group) and the VTF dose groups were established by injecting Aβ_(25–35)_ (Sigma, St. Louis, United States) with a microinjector into the bilateral hippocampal CA1 region (5 μl per side). The region was located according to the brain stereotaxic atlas. Sterile saline was injected as normal control. The needle was left in place for 5 min, and then the wound was sutured.

### Animal grouping

According to the random number table, the rats were randomly divided into 6 groups of 15 rats each:

- control group
- model group
- donepezil (Eisai China Inc., Shanghai, China) group (0.5 mg/kg)
- low-dose VTF group (50 mg/kg)
- medium-dose VTF group (150 mg/kg)
- high-dose VTF group (300 mg/kg)

Intragastric administration was performed once daily (1.0 ml/100 g) for 14 consecutive days. Rats in the control group and the model group received equal volumes of saline.

### Morris water maze test

Rats were subjected to double-blind water maze training on the 13th day of administration. After 6 days, rats were subjected to the space exploration experiment of the Morris water maze. The escape platform was removed and rats were pooled from the original position and allowed to swim for 90 s. The percentage of swimming time spent in the third quadrant within 90 s and the number of times the rats crossed the effective area were recorded.

### Sample collection

The rats were anesthetized with 10% chloral hydrate (0.4 ml/100 g). Then their (*n* = 9) brains were perfused and fixed for 24 h in 4% paraformaldehyde for immunohistochemical staining. The bilateral hippocampi were harvested on ice from the remaining brain tissue of the rats (*n* = 6), placed into a labeled freezing tube, rapidly frozen with liquid nitrogen and stored at − 80 °C.

### Detection of ChAT and AChE

Researchers cut a small piece cut from the hippocampus tissue and added it to the pre-cooled saline, then added 9**×** the volume of cold saline homogenate. They centrifuged this mixture for 10 min at 2500 rpm at 4 °C, then harvested the supernatant as the sample to be tested. ChAT and AChE in the hippocampus was determined using a ChAT and AChE assay kit.

### Immunohistochemistry staining

The sections that had been conventionally embedded in paraffin were dewaxed in water, then subjected to microwave antigen retrieval in a 3% hydrogen peroxide solution to inactivate endogenous peroxidase activity. After blocking with 10% goat serum, researchers incubated the sections at 4 °C overnight, then added horseradish peroxidase labeled as secondary antibodies of IgG. They then developed the sections with a 3,3′-diaminobenzidine (DAB) chromogenic reagent. The first antibody was diluted by a ratio 1:100 for BDNF, SYT1 and CREB (all 3 from Abcam Inc., Cambridge, UK). Neutral gum sealing piece. An immunohistochemical section of the positive staining neurons in the hippocampi was observed or collected images under a microscope. Results: Nuclear and cytoplasmic cells that were stained brown were positive.

### Western blot

Researchers took a sample of rat hippocampus tissue and added 400 μl phenylmethanesulfonyl fluoride (PMSF) to the liquid. After centrifugation, they harvested the supernatant with a nuclear protein extraction kit. The concentration of protein was determined by BCA method. The total protein and nuclear protein extract was mixed with a 5**×** protein sample buffer at a 4:1 ratio. After cooling in the polyacrylamide gel electrophoresis (PAGE) sample hole, the mixture was boiled in a water bath for 10 min.

Researchers performed ECL Western blot to detect protein expression of BDNF, SYT1, CREB and p-CREB (Abcam Inc., Cambridge, UK), repeating the experiments 3 times. Techniques used were dry film, film scanning, and band scan analysis of the target band gray value. For BDNF and SYT1, glyceraldehyde 3-phosphate dehydrogenase (GAPDH; Goodhere, Hangzhou, China) was used as the internal control; for p-CREB and CREB, lamin B1 was used as the internal control. Film transfer conditions were as follows:

- BDNF: 200 mA, 50 min
- GAPDH: 200 mA, 90 min
- SYT1, CREB, p-CREB, lamin B: 200 mA, 120 min

### Real-time quantitative PCR

Total RNA was extracted using TRIzol Reagent (ThermoFisher Scientific., Carlsbad, United States) according to the manufacturer’s instructions. Researchers performed quantitative reverse transcription PCR (RT-qPCR) with ABI 7900 PCR (Applied Biosystems, CA, United States). Primes used in RT-qPCR are listed in Table 1. Complementary DNA (CDNA) was diluted 10**×**. The CREB gene amplification system is the same as for GAPDH. The amplification conditions were as follows (40 cycles):

- Pre-denaturation: 50 °C, 2 min
- Denaturation: 95 °C, 10 min
- Annealing: 95 °C, 30 s
- Extension: 60 °C, 30 s

**Table 1:** The primer sequence

Name	Sense	Anti-sense	Size(bp)
CREB	5′- TCAGCCGGGTACTACCATTC-3′	5′- CCTCTCTCTTTCGTGCTGCT-3′	218
GAPDH	5‘-ACAGCAACAGGGTGGTGGAC-3’	5′- TTTGAGGGTGCAGCGAACTT − 3	253

Researchers repeated the experiment 3 times. They confirmed specificity of all PCR products by melting curve analysis and calculated the expression using 2^-∆∆Ct^.

### Statistical analyses

Researchers analyzed the data using IBM SPSS Statistics software, version 22.0. Data was expressed as the mean ± the standard error of the mean (SEM). The comparison of means among several groups was analyzed with One-Way ANOVA. When the deviations were equal, it was chosen to use LSD inspection; when the deviations were different, it was chosen to use Tamhane’sT2 for the inspection. A *p*-value of less than 0.05 was considered statistically significant.

## Results

### Effect of VTF on memory learning in AD model rats

The effect of VTF on memory learning ability of AD model rats was detected by water maze test. The escape latency of the rats in the model group increased significantly in comparison with the control group (36.86 ± 13.36 s in model group vs 25.39 ± 8.58 s in control group, *p* < 0.01, Table 2). Compared with the model group, the escape latency of medium, and high-dose VTF groups reduced (29.58 ± 7.53 s and 28.05 ± 4.74 s in VTF groups vs 36.86 ± 13.36 s in model group, *p* < 0.05, Table 2). In the space exploration experiment, the number of times the platform was crossed by the rats in model group decreased significantly compared with the control group (3.08 ± 1.19 times in model group vs. 7.13 ± 1.64 times in control group, *p* < 0.01, Table 3). While, the number of crossing times of the donepezil (6.54 ± 3.02 times) significantly increased than those of the model group (*p* < 0.01, Table 3), and the high-dose VTF groups (4.86 ± 1.56 times) also increased than those of the model group (*p* < 0.05, Table 3). These results suggest that donepezil and VTF effectively improves the learning and memory ability of AD rats.

**Table 2:** The change of average of the escape latency in each group rats ($\overline{\chi}\pm s$, *n* = 15)

Group	The average incubation period(s)
Control group	25.39 ± 8.58
Model group	36.86 ± 13.36^**#**^
Donepezil group	30.25 ± 9.31
Low-dose VTF group	31.72 ± 5.71
Medium-dose VTF group	29.58 ± 7.53^*^
High-dose VTF group	28.05 ± 4.74^*^

^#^The *p*-value was less than 0.01 compared with the control group

*The *p*-value was less than 0.05 compared with the model group

**Table 3:** The effective area entry times of rats in each ($\overline{\chi}\pm s$, n = 15)

Group	The effective area entry times
Control group	7.13 ± 1.64
Model group	3.08 ± 1.19^#^
Donepezil group	6.54 ± 3.02**
Low-dose VTF group	3.00 ± 1.58
Medium-dose VTF group	2.30 ± 1.95
High-dose VTF group	4.86 ± 1.56*

^#^The *p*-value was less than 0.01 compared with the control group

*The *p*-value was less than 0.05 compared with the model group

**The *p*-value was less than 0.01 compared with the model group

### Immunohistochemical results

**Fig. 1:**
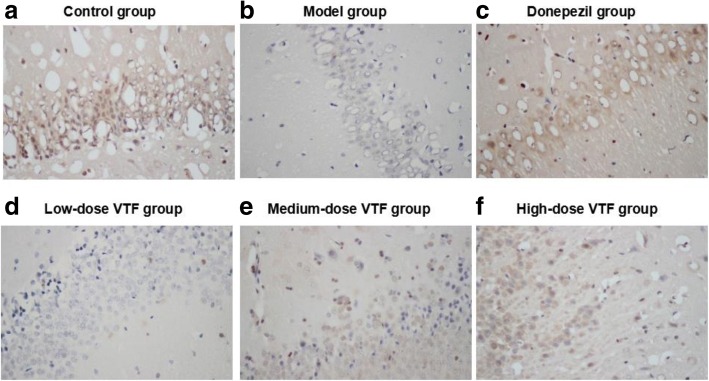
Expression of CREB in the hippocampus of rat in different groups (*n* = 9) with high (Immunohistochemistry × 400) magnification were shown. Hippocampus tissues were collected from control group (**a**), model group (**b**), donepezil group (**c**), low-dose VTF group (**d**), medium-dose VTF group (**e**), and high-dose VTF group (**f**)

The hippocampus of rats was selected as the study area. The results showed that in CREB-positive cells, CREB was expressed mainly in the cells’ nuclei (400**×** magnification). The CREB immunoreactive cells in the hippocampi of rats in the model group were significantly lighter than those in the control group, while those in the VTF groups were significantly darker than those in the model group (Fig. 1).

**Fig. 2:**
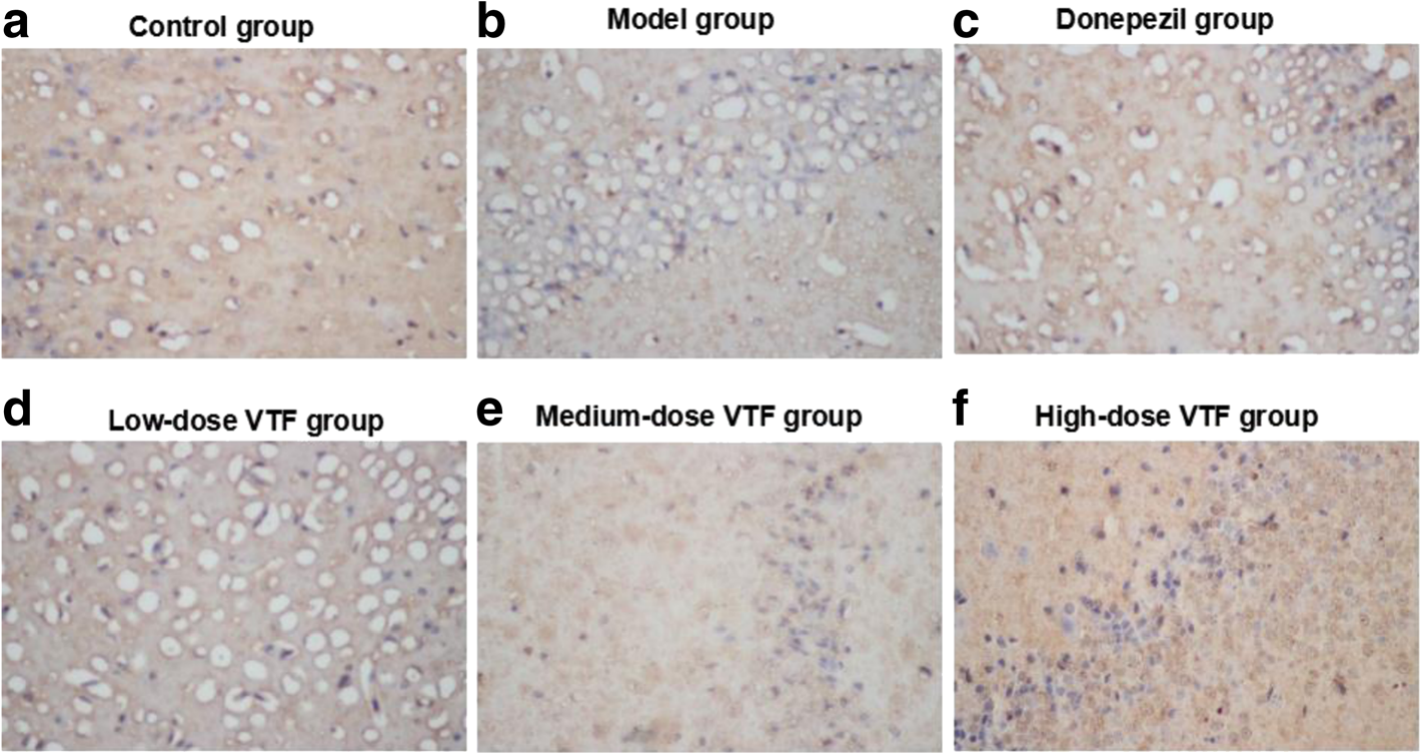
Expression of BDNF in the hippocampus of rat in different groups (*n* = 9) with high (Immunohistochemistry × 400) magnification were shown. Hippocampus tissues were collected from control group (**a**), model group (**b**), donepezil group (**c**), low-dose VTF group (**d**), medium-dose VTF group (**e**), and high-dose VTF group (**f**)

**Fig. 3:**
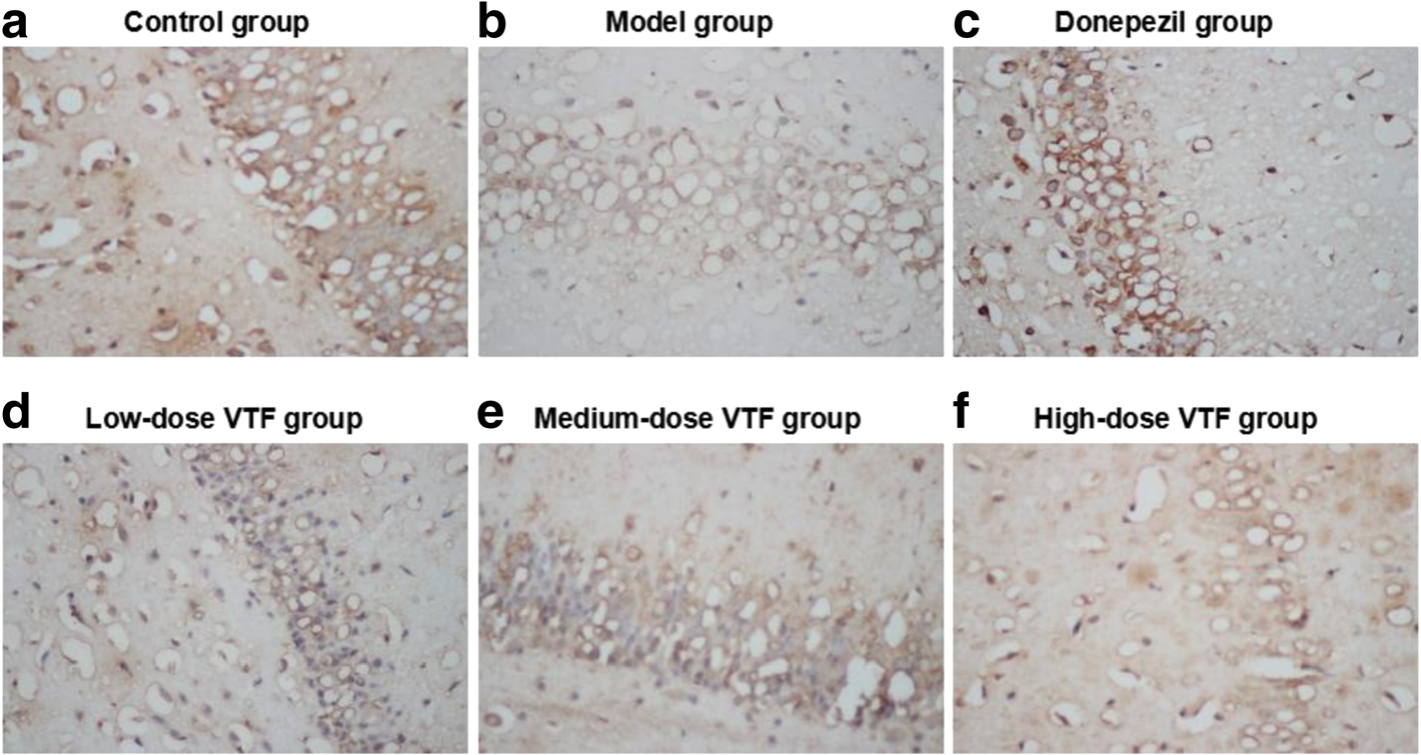
Expression of Syt-1 in the hippocampus of rat in different groups (*n* = 9) with high (Immunohistochemistry × 400) magnification were shown. Hippocampus tissues were collected from control group (**a**), model group (**b**), donepezil group (**c**), low-dose VTF group (**d**), medium-dose VTF group (**e**), and high-dose VTF group (**f**)

Researchers observed immunohistochemistry results of BDNF and SYT1 by optical microscope (400**×** magnification). In BDNF- and SYT1-positive cells, expression occurred mainly in cytoplasm. The BDNF- and SYT1-positive cells in the hippocampi of rats in the model group were lighter than those in the control group, while those in the VTF groups were darker than those in the model group (Figs. 2 and 3).

### Western blot results

**Fig. 4:**
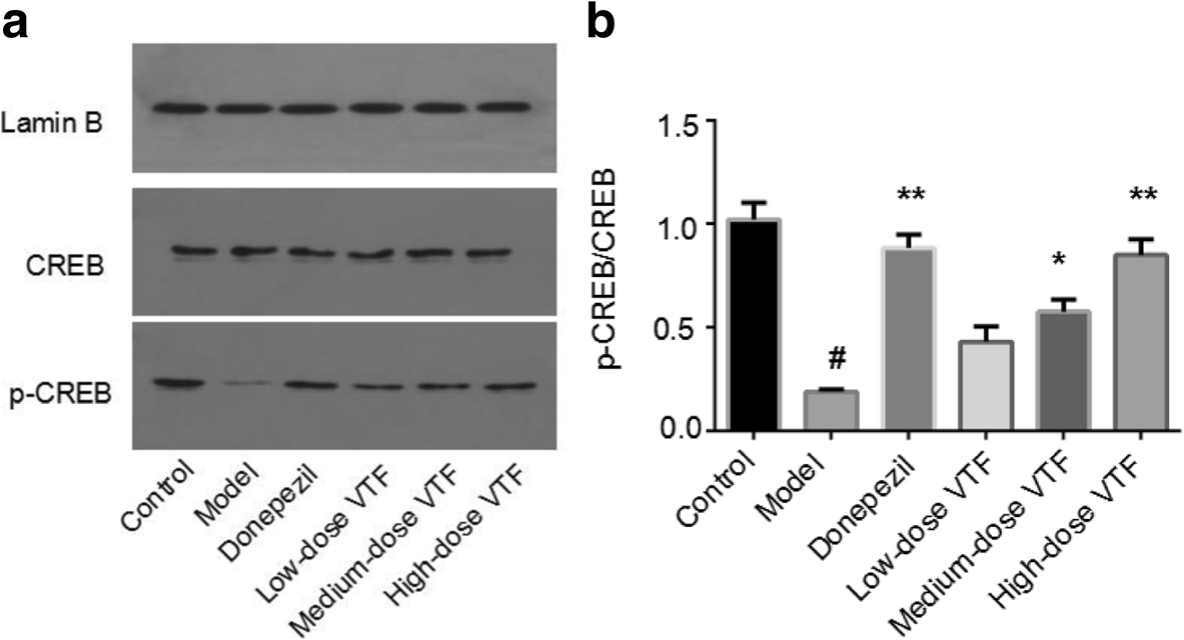
Effect of VTF on the expression of CREB and p-CREB at protein level. **a** The expression of CREB and p-CREB detected by western blot. **b** Relative expression expressed as the mean ± SEM (*n* = 6). # The *p*-value was less than 0.01 compared with the control group. * The *p*-value was less than 0.05 compared with the model group. ** The *p*-value was less than 0.01 compared with the model group

In this study, researchers detected the expression of CREB and p-CREB protein by Western blot. They found that, compared with the control group (1.02 ± 0.08), the ratio of p-CREB/CREB in the model group (0.19 ± 0.01) were decreased by a statistically significant amount (*p* < 0.01). Compared with the model group, the p-CREB/CREB values in the medium-dose and high-dose VTF groups increased by different degrees (3.1 fold and 4.5 fold), both of which were statistically significant (*p* < 0.05 or *p* < 0.01, Fig. 4). These results suggest that VTF could enhance the expression of p-CREB, reduce the loss of hippocampal neurons in AD rats, and improve learning and memory ability in AD rats.

**Fig. 5:**
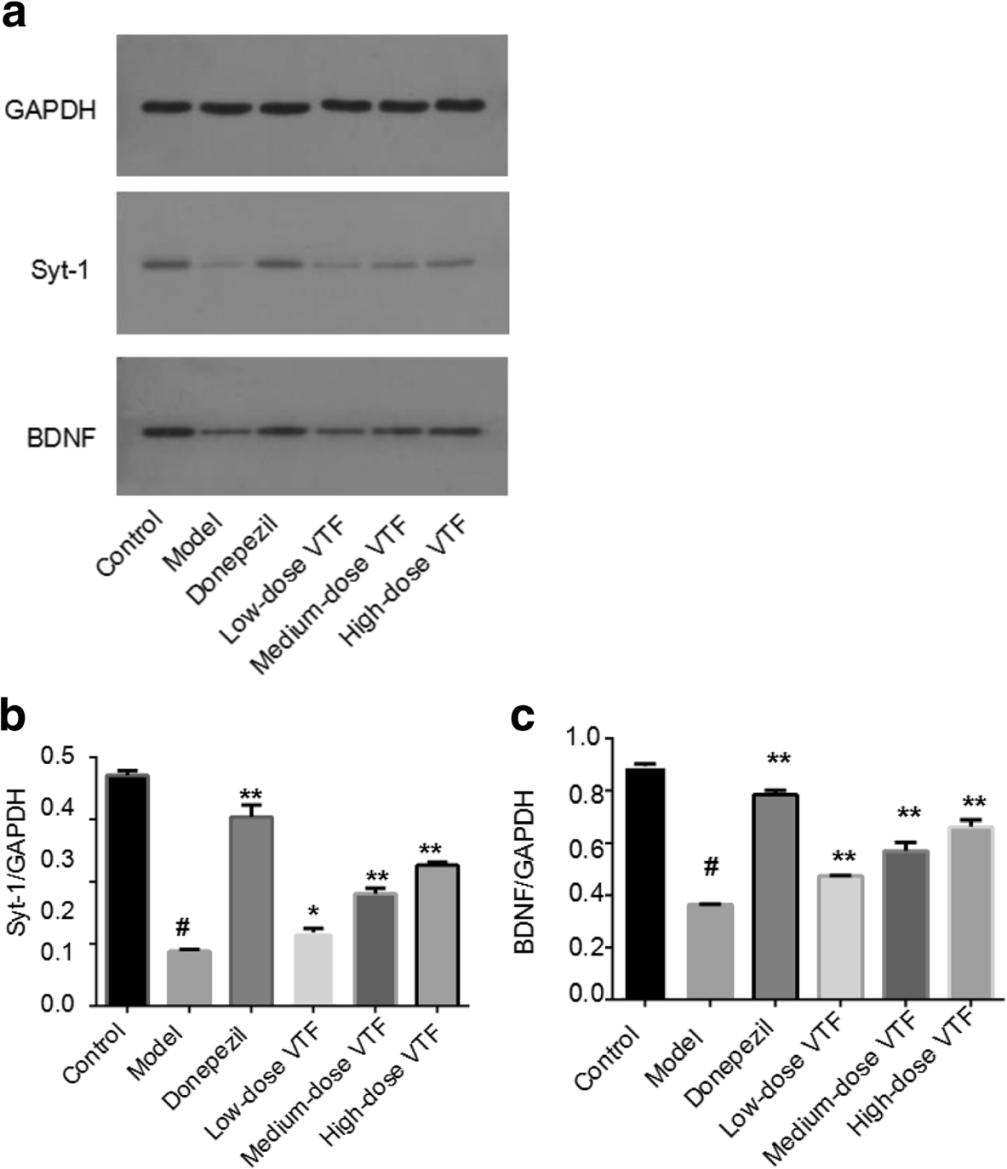
Effect of VTF on the expression of SYT1 and BDNF at protein level. **a** The expression of SYT1 and BDNF detected by western blot. **b** The expression of SYT1 at protein level (**c**) The expression of BDNF at protein level. Data were expressed as the mean ± SEM (*n* = 6). # The *p*-value was less than 0.01 compared with the control group. * The *p*-value was less than 0.05 compared with the model group. ** The *p*-value was less than 0.01 compared with the model group

To investigate how VTF affected the synaptic plasticity proteins BDNF and SYT1, the researchers also used Western blot to detect the expression of these proteins. They found that the protein levels of both BDNF and SYT1 in the model group were significantly lower than those in the control group (*p* < 0.01, Fig. 5), which suggests that decreases in BDNF and SYT1 may be involved in the degradation of nerve function in AD. Compared with the model group, the expression of BDNF in the VTF groups was significantly increased (*p* < 0.01, Fig. 5).and the protein expression was positively correlated with the dose of VTF. The results also showed that the VTF significantly improved the protein expression of SYT1 (0.12 ± 0.01 in low-dose VTF group vs 0.09 ± 0.02 in model group, *p* < 0.05. 0.18 ± 0.01, 0.23 ± 0.005 in the medium-dose and high-dose VTF groups vs 0.09 ± 0.02 in model group, *p* < 0.01, Fig. 5). These results suggest that VTF can upregulate the expression of BDNF and SYT1 proteins. Therefore, one can explain the effect of VTF on the cognitive impairment of AD rats.

### RT-qPCR results

**Fig. 6:**
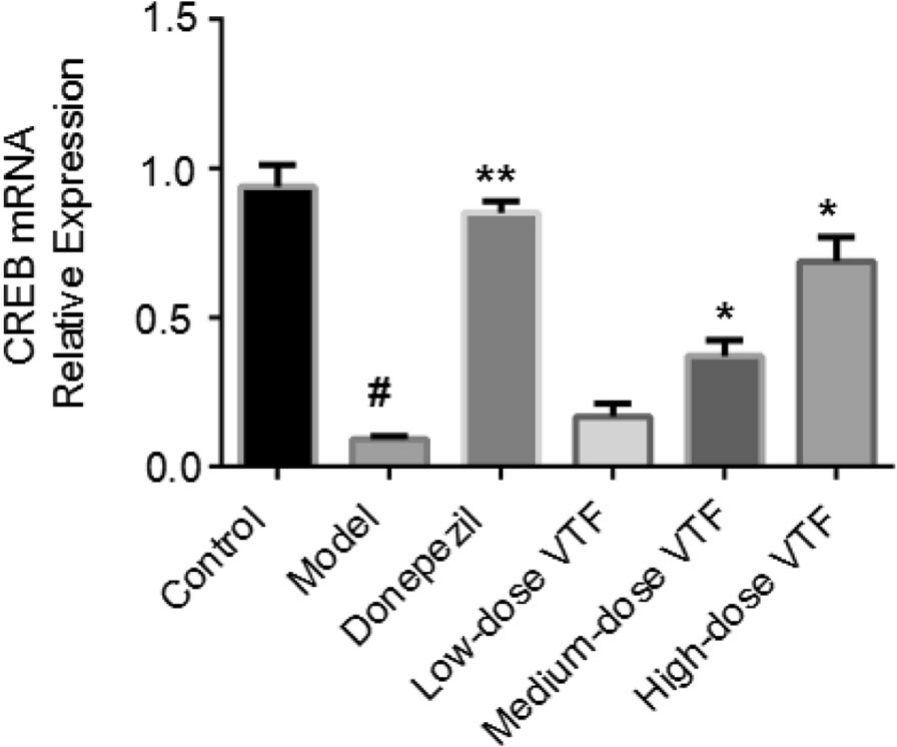
Effect of VTF on the expression of CREB at mRNA level in the hippocampus of rats. Data were expressed as the mean ± SEM (*n* = 6). # The *p*-value was less than 0.01 compared with the control group. * The *p*-value was less than 0.05 compared with the model group. ** The *p*-value was less than 0.01 compared with the model group

To further verify the role of VTF in regulating CREB from the gene level, researchers extracted total RNA from the rat hippocampi and performed RT-qPCR. As shown in Fig. 6, the mRNA levels of CREB in the hippocampi of the model group (0.09 ± 0.01) were much lower than those in the control group (0.94 ± 0.07), and the difference was statistically significant (*p* < 0.01). The mRNA levels of CREB in the donepezil (0.85 ± 0.04) group were significantly greater than those in the model group, and the difference was again statistically significant (*p* < 0.01). The mRNA levels of CREB in the medium-dose (0.37 ± 0.05) and high-dose VTF group (0.69 ± 0.08) were significantly greater than those in the model group (0.09 ± 0.01, *p* < 0.05). These results confirm those of the Western blot by demonstrating that VTF enhanced the activation of CREB.

### Comparison of ChAT and AChE activity in rat hippocampi

**Fig. 7:**
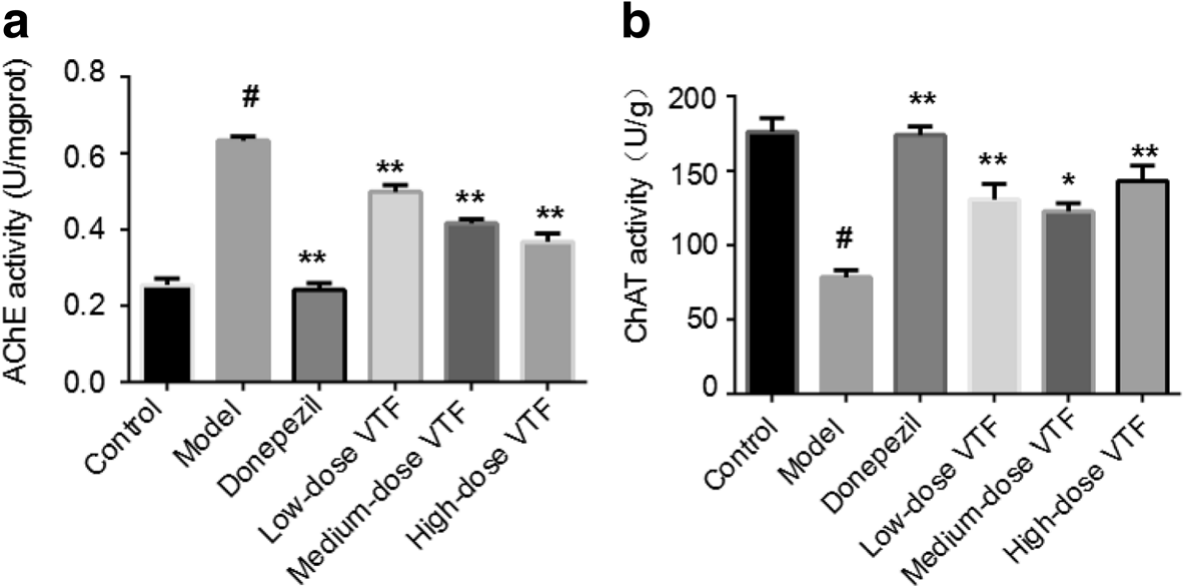
Changes in the activity of AChE and ChAT in the hippocampus. **a** The activity of AChE in the hippocampus. **b** The activity of ChAT in the hippocampus. Data were expressed as the mean ± SEM (n = 6). # The *p*-value was less than 0.01 compared with the control group. * The *p*-value was less than 0.05 compared with the model group. ** The *p*-value was less than 0.01 compared with the model group

The activity of AChE in the hippocampi of rats in the model group (0.63 ± 0.01) was significantly higher than those of the control group (0.25 ± 0.02, *p* < 0.01). Compared with the model group, AChE activity decreased very significantly in the VTF groups (*p* < 0.01), and the change was positively correlated with the dose of VTF. The activity of ChAT in the hippocampi of rats in the model group (78.65 ± 4.58) was significantly lower than in those of the control group (176.03 ± 9.2, *p* < 0.01). Compared with the model group, ChAT activity increased significantly in the medium-dose VTF group (122.91 ± 5.49, *p* < 0.05) and very significantly increased in the high-dose (143.45 ± 10.2) and low-dose VTF groups (130.91 ± 10.57, *p* < 0.01). The differences were statistically significant (Fig. 7).

## Discussion

### Effect of VTF on CREB

The material basis of learning and memory is hippocampal synaptic plasticity and long-term potentiation (LTP) [14, 15]. The biological activity of CREB protein is mainly regulated by phosphorylation of the Ser133 and Ser142 sites. Phosphorylation of the Ser133 site could activate the transcription of downstream genes, regulate nerve excitability and play a role in neural protection.

The role of CREB is becoming more and more important in AD research. The level of CREB phosphorylation and the expression of downstream target genes related to synaptic plasticity and learning and memory (e.g., BDNF and SYT1) has been shown to be inhibited in amyloid precursor protein (APP) transgenic mice, demonstrating that CREB has an important relationship to the pathophysiology of AD [16–18]. To explore the mechanism of VTF on synaptic plasticity in AD rats, researchers detected CREB protein changes in rat hippocampi using immunohistochemistry, Western blotting and qRT-PCR. The results showed that the p-CREB and CREB values in the hippocampi of rats of the AD model group decreased significantly, as did expression of CREB mRNA, which may be due to the direct effect of Aβ_(25–35)_. The expression of CREB mRNA and the ratio of p-CREB to CREB were positively correlated with the dose of VTF. The downregulation of CREB phosphorylation is therefore closely related to the decline of learning and memory ability in patients with AD. The results also showed that VTF in each dose group significantly increased the level of CREB phosphorylation. From this, the researchers speculate that VTF may maintain the balance of calcium homeostasis, increase the content of phosphorylated CREB and regulate synaptic plasticity by reducing the deposition of Aβ in rat hippocampi. VTF, therefore, plays a role in the prevention of AD.

### Effects of VTF on BDNF and SYT1

The expression of BDNF in learning- and memory-related brain regions, especially in the hippocampus, is related to learning and memory [19–22]. The results showed that expression of BDNF in the brains of AD patients was decreased and that the expression of Aβ could downregulate the expression of BDNF, which could affect the learning and memory functions [23, 24]. On the other hand, the regulation of BDNF expression could relieve the neurotoxicity of Aβ, enhance synaptic plasticity and improve cognitive impairment [25]. In this study, compared with the control group, the expression of BDNF in the hippocampus of the model group was decreased, suggesting that this decrease may be involved in the degradation of nerve function in AD. In each dose group, the hippocampal expression of BDNF was increased and positively correlated with the dose of VTF. BDNF not only promoted neuronal regeneration but also regulated synaptic plasticity [26]. Therefore, VTF can improve the learning and memory functions in AD rats by upregulating BDNF expression.

The study also showed that brain levels of SYT1, which affects the release of neurotransmitters, were also significantly reduced in patients with AD [27, 28]. By injecting Aβ_(25–35)_ into the hippocampus, researchers were able to induce cognitive dysfunction in rats. At the same time, the expression of SYT1 protein decreased, suggesting that this decrease might also be related to the degeneration of nerve function in AD. After treatment with VTF in AD rats, the expression of SYT1 in the hippocampus was increased, which was positively correlated with the dose of VTF. These results suggest that VTF could improve learning and memory function in AD rats by upregulating SYT1 expression.

### Effect of VTF on cholinergic system

The study found that cognitive dysfunction was closely related to the decrease of the activity of cholinergic transmitters and that ACh was an important neurotransmitter in the cholinergic system governing learning and memory. Activities of ACh and ChAT in the serum of AD patients is decreased [29, 30]. Because ACh is unstable, AChE and ChAT are the most important indicators of the central cholinergic system’s status. The results showed that VTF can inhibit the activity of AChE and enhance that of ChAT. The level of acetylcholine (ACh) was indirectly increased, meaning that the protective effect of VTF on AD might be related to the enhancement of cholinergic nerve function.

The researchers found that BDNF was closely related to the cholinergic system. BDNF can promote the production of ACh and the development of cholinergic neurons in rat hippocampi; thus, it could protect cognitive function [31]. It has been reported that when ACh receptors on PC12 cells were activated, the CaM(Calmodulin)/CaMKII(Calcium/Calmodulin-dependent protein kinase II)/CREB signaling pathway was activated [32]. CREB being the major regulatory factor of BDNF, CREB phosphorylation could regulate BDNF transcription. Therefore, VTF is likely to enhance the level of ACh by inhibiting AChE activity and enhancing ChAT activity, thereby activating the ACh receptor. CREB phosphorylation further influenced BDNF synthesis. What is the intrinsic connection between BDNF and ACh? What role do they play in the regulation of the CaM/CaMKII/CREB signaling pathway? All these questions need further study.

## Conclusion

VTF can enhance the activity of p-CREB, BDNF and SYT1 in the hippocampus of rats, thereby promoting the synaptic plasticity and indirectly affecting the expression of cholinergic neurotransmitters. This may be one of the mechanisms of VTF protection in AD rats.

## Abbreviations

AChE: Acetylcholinesterase
AD: Alzheimer disease
APP: Amyloid precursor protein
BDNF: Brain-derived neurotrophic factor
CaM: Calmodulin
CaMKII: Calcium/Calmodulin-dependent protein kinase II
ChAT: Choline acetyltransferase
CREB: cAMP response element-binding protein
DAB: Diaminobenzidine
GAPDH: Glyceraldehyde 3-phosphate dehydrogenase
LTP: Long-term potentiation
mRNA: Messenger ribonucleic acid
PAGE: Polyacrylamide gel electrophoresis
PMSF: Phenyl methanesulfonyl fluoride
qRT-PCR: Real-time fluorogentic quantitative PCR
SD: Sprague-Dawley
SPF: Specific-pathogen-free
SYT1: Synaptotagmin-1
VTF: *Vitis vinifera* L
